# Supplementary material for: Are carbon emissions trading and green financial instruments synergistic? -Comprehensive quantitative research based on content analysis
Source: PLoS One. 2024 Mar 7;19(3):e0298601. doi: 10.1371/journal.pone.0298601 (PMC10919601; doi:10.1371/journal.pone.0298601)
Supplement: S1 Data — (ZIP) [file pone.0298601.s001.zip › Data/地级市Z_F工作报告.zip!/░▓╗╒/╬▀║■╩╨/└┤╫╘╣1⁄2╓┌║┼í╛┬φ┐╦╩2╛▌═°í┐_2011.docx]

——2011年1月8日在芜湖市第十四届人民代表大会第五次会议上

市长 杨敬农

各位代表：

现在，我代表市人民政府，向大会作政府工作报告，请予审议，并请市政协各位委员和其他列席人员提出意见。

一、厚积薄发、成就辉煌的奋进历程

过去的一年，全市人民在中共芜湖市委的坚强领导下，深入落实科学发展观，积极应对宏观环境变化，加快推进经济结构调整和发展方式转变，保持了经济社会又好又快发展，圆满完成了市十四届人大四次会议确定的各项任务，全面实现了“十一五”规划目标。

——经济总量跃上千亿元台阶，发展速度进一步加快。全市实现地区生产总值1080亿元（预计数，下同），比上年增长17.5%；人均地区生产总值突破7000美元。全部工业销售收入达2200亿元，增长35%。实现财政收入200.7亿元，增长35.8%，其中地方财政收入94.8亿元，增长36.6%。

——固定资产投资突破千亿元，有效需求进一步扩大。全社会固定资产投资超过1200亿元，增长33.2%以上。社会消费品零售总额287亿元，增长19.3%。进出口总额25亿美元，增长75.1%，其中出口16.7亿美元，增长86.5%，创历史新高。

——城乡居民收入保持稳定增长，人民生活进一步改善。城市居民人均可支配收入18600元，增长11%；农民人均纯收入7410元，增长10%；城乡居民人均储蓄存款2.06万元，增长16.6%；城镇新增就业6万人，城镇登记失业率控制在4%以内。

——节能减排目标全面实现，生态环境进一步优化。完成省政府下达的节能减排任务。我市在2009年度全国城市环境综合整治定量考核中，名列全省第一。

一年来，我们主要做了以下几个方面的工作：

（一）围绕加速崛起目标，巩固经济回升向好势头。一是推进项目建设。全年在建投资亿元以上非房地产项目275个，十亿元以上项目37个，百亿元以上项目3个。芜湖电厂五期，三安光电、德豪润达光电、集瑞联合重工、玉柴联合动力、美的洗涤电器和厨卫电器、新联船厂新厂等项目一期建成投产，规模以上工业企业比上年净增397户。新兴铸管扩建、恒安纸业、双汇食品等项目开工建设。宁安城际铁路、合福客运专线、芜申运河、芜雁高速公路工程全面推进。核电项目列入国家中长期规划。二是完善企业服务。兑现企业税收优惠政策资金34亿元。减缴、核销企业社保费2.8亿元，缓缴社保费3.3亿元，发放社保补贴1.3亿元。35户工业企业列入了“小巨人”培育计划。坚持经济运行调度制度，加大企业帮扶力度，着力解决融资难等问题，小企业贷款余额272亿元，比年初增加196亿元。三是努力扩大内需。落实家电、汽车、摩托车下乡和“以旧换新”等促进消费政策，财政补贴9186万元。大力培育文化、旅游等消费热点。四是增强县区发展活力。市级调度资金102亿元，支持县区发展。调整市与区财政体制，当年新增区级可用财力4.2亿元。五是夺取防汛抗洪重大胜利。面对近十年来长江最高水位、最大汛情，科学调度，全力以赴抗洪抢险，千亩以上圩口无一溃破，市区无明显内涝。

（二）调整优化经济结构，构建现代产业体系。深入实施工业强市、三产兴市战略，着力推进产业优化升级。汽车及零部件、材料、电子电器三个支柱产业实现总产值1640亿元，增长42.6%。汽车产量68万辆，空调产量突破700万台。节能环保、装备制造、电子信息、生物医药四个战略性新兴产业完成投资180亿元，增长66%；实现产值380亿元，增长46.8%。引进了兴业银行、江阴农商行等4家域外金融机构，繁昌建信村镇银行开业，扬子银行注册资金增加到10亿元。皖江物流产业投资基金获批设立，首期募集50亿元。新增小额贷款公司14家、证券保险机构2家、典当机构2家、担保机构72家。组建了皖江股权交易所。金融机构本外币存款余额1260亿元、贷款余额1040亿元，分别增长29.7%、28.4%；域外金融机构贷款170亿元。神剑股份、长信科技成功上市，2家企业完成上市申报，芜湖港成功重组，市建投、经济技术开发区建投公司共发行企业债券30亿元，海螺、奇瑞共发行中期票据68亿元，全市在资本市场筹资超过170亿元。出口加工区、芜湖港、裕溪口等物流园区建设加快，港储30万标箱码头工程竣工，新增第三方物流企业6家，安得物流成为全省首个国家5A级物流企业。商品博览城、果品冻品水产批发市场等14个专业市场建设加快，芜湖长江船舶交易市场交易量位居全国第一。我市被评为全国流通领域现代物流示范城市。方特梦幻王国主题公园建成开园。建成和在建的四星级标准以上饭店13家，其中五星级7家。旅游总收入76亿元，增长24.9%。服务外包企业新增52家，服务外包执行金额达5000万美元，增长3.4倍。成功举办了科博会、汽博会等规模会展18个，我市被评为中国十佳会展城市。推广超级杂交稻41万亩，实施12万亩国家优质油菜生产基地项目，新增省级农业产业化龙头企业13家。南陵县跻身国家首批现代农业示范区行列，大浦园区被批准为国家农业科技园区。

（三）着力转变发展方式，提升城市核心竞争力。全面推进国家技术创新工程试点和合芜蚌自主创新综合试验区建设，制定了鼓励风险投资机构推动领军人才创新创业、扶持光电产业发展等政策，市财政安排7.5亿元支持企业技术创新，芜湖高新技术产业开发区升格为国家级。全市获得省级以上科技成果36项，新增省级创新型（试点）企业10家、高新技术产品111个；专利申请量7600件、授权量3500件，分别增长73.5%、30%；新增国家级博士后科研工作站2家、省工程技术研究中心10家、省级企业技术中心18家、省部共建国家重点实验室培育基地和省级技术转移示范机构各1家。大力实施商标品牌战略，“精诚”、“奔牛”、“同福”被认定为中国驰名商标，全市新增省著名商标12件、省名牌产品12件、核准注册商标1350件。奇瑞建成了亚洲规模最大的汽车试验技术中心和汽车节能环保国家工程实验室。华东光电研究所特种显示国家工程实验室获批建设。中天印染公司技术中心被认定为国家级企业技术中心。我市成为国家知识产权工作示范城市、国家节能与新能源汽车高新技术产业化基地。

强化节能减排工作责任制，加强重点企业和行业监管。实施了85个节能项目，奇瑞5款新能源车型入选国家新能源汽车推荐目录，9个项目列入国家十大重点节能工程、循环经济和资源节约重大示范项目。安装节能路灯1.4万盏，节电50%以上；建筑节能施工执行率达100%，市区新型墙体材料应用比重达98.5%。我市成为全国可再生能源建筑应用示范城市。加快淘汰落后产能，关停小火电机组2台、立窑水泥生产线26条、粘土砖瓦企业15家、非煤矿山23家。建成了朱家桥二期、城南一期和南陵县污水处理工程，新增污水处理能力24万吨。实施“蓝天工程”，整治炉窑灶678台。

（四）大力统筹城乡发展，加快推进城市化进程。坚持高起点规划、大手笔建设，出台了加快城市化进程的实施意见和配套措施。完成了市县两级土地利用总体规划省级评审，编制了城市总体规划纲要和两江两湖（长江、青弋江、龙窝湖、黑沙湖）地区概念性规划等专项规划，完成了板城垾水系综合整治、大阳垾湿地公园、三县四区发展战略等规划方案。城市基础设施建设完成投资140多亿元。长江中路南段高架桥、神山路跨扁担河桥、袁泽桥西幅桥和北京中路拓宽改造等工程建成通车。205国道等路面改善工程完工，新建改建农村公路234公里。新建、改造了220千伏供电线路19条。完成了6座病险水库除险加固工程，解决了13.9万农村人口饮水安全问题。推广农村信息化综合信息服务试点成果，宽带数据网实现市域全覆盖。新增投放客运出租车480辆、公交车70辆，整治了“五小”车辆，取缔残疾人机动轮椅车非法营运。开展市容整治巩固年活动，大力实施美化、绿化、亮化工程，整治了滨江南路等11条道路架空杆线，改造了44个老旧居民小区、60万平方米棚户区和北京路、利民路等沿街立面，新增赭山公园、中央公园等处绿化面积480万平方米，新增营造林7.95万亩，改扩建亮化项目100个，拆除违法建设13.6万平方米。发挥数字化城管平台作用，开通了城管服务热线，提高城市管理水平。

（五）不断深化改革开放，增强发展活力和动力。一是推进统筹城乡综合配套改革。基本完成了农村集体土地确权发证工作。推进农村土地承包经营权流转和集体林权制度配套改革，流转耕地21万亩、林地6万亩。实施土地整治整村推进工作，开发复垦整理新增耕地9450亩，盘活存量用地2367亩，收回低效闲置土地1189亩。二是推进医药卫生体制改革。实施了全国公立医院改革试点和基层医药卫生体制综合改革，组建了市三大医疗集团，整合区级医院，创新管理体制机制。医药分开改革向县级延伸，推行国家基本药物制度，政府举办的基层医疗卫生机构全部实现药品零差率销售，减轻群众药费负担8570万元。三是推进财政和国有资产监管改革。推进税源专业化管理试点，强化了税费征管。清理政府性债务和融资平台债务，制定了市直事业单位贷款、财政结转结余资金管理办法。开展了财政资金绩效评估试点。实施了行政事业单位资产信息化管理，加强国有资产监管。出台了商品房验收和政府投资建设项目第三方审计办法。四是推进事业单位收入分配制度改革。完成了公共卫生和基层医疗卫生人员绩效工资改革，预发了其它事业单位工资（生活）性补贴。五是大力发展民营经济。召开了全市创业富民大会，出台一系列政策，推进全民创业。全市登记个体工商户、私营企业达7.27万户，增长9.3%；新开业个体工商户1.26万户、私营企业3587户。

全面启动皖江城市带承接产业转移示范区建设。经济技术开发区基础设施进一步完善，长江大桥开发区完成了新区总体规划和循环经济园区、综合保税区规划，省级孙村经济开发区获批筹建。制定了代理招商管理办法，组织参加了中博会、徽商大会等重大招商活动，在京、沪、闽、粤等地举办了14次投资环境推介会，选派了第二批市直驻点招商人员，推进市直小分队招商向专业招商转变。加强与中央企业、全国知名民营企业对接，8个央企合作项目开工建设，格力电器、德尔电器、南翔万商等一大批项目成功落户。全年实际利用外资7.4亿美元，增长17.5%；实际利用内资1150亿元，增长71.2%。新增进出口经营权企业230家、发生实绩企业90家。港口货物吞吐量达6600万吨，增长15.6%。外事、侨务、对台、外经等工作取得新进展，与亳州结对合作取得初步成效，与奥地利格拉茨市缔结为友好城市。

（六）努力保障改善民生，维护社会和谐稳定。大力实施38项民生工程，总投入36.8亿元。完成农村劳动力转移培训2.8万人，农民工技能培训1.3万人。全市最低月工资标准从540元提高到680元。城镇职工基本养老、失业、医疗、工伤、生育保险参保人数共达153.6万人次，增长8%。市属三县全部纳入了国家新农保政策试点。退休人员养老金月人均增加127元，失业保险金月人均增加91元。10万城乡低保对象应保尽保，对困难群众的医疗救助、临时救助水平进一步提高。改扩建敬老院15所，新增床位1200张。五保对象分散供养和集中供养标准分别达到年人均1880元、2570元以上。新型农村合作医疗参合人数101.9万人、参合率98.8%，城镇居民基本医疗保险参保77万人。新增131个单位、1.7万名职工建立了住房公积金制度。加强房地产市场调控，完成房地产开发投资280亿元，增长35.4%；房屋竣工270万平方米，增长24%。安置房在建665.3万平方米、累计竣工619.2万平方米。廉租房在建87.1万平方米、竣工40.9万平方米。11374户居民享受廉租住房保障，其中1883户享受实物配租。义务教育阶段学校标准化建设稳步推进，完成了市区中小学校舍安全工程年度目标和三县农村学校旱厕改造任务。改革职业教育办学模式，毕业生双证率、就业率均达98%。扩大优质高中资源，高中阶段毛入学率提高到84.8%。市一中新校区、安徽中加学校即将投入使用，新增民办教育机构43个。弋矶山医院、市二院、市四院门诊住院楼投入使用，新增病床1500张。市中医院门诊医技住院楼主体封顶。建成1个标准化镇卫生院、244个标准化村卫生室。徽商博物馆建成开馆。4个镇综合文化站、38个自然村广播电视“村村通”工程、79个农家书屋、50个村农民体育健身工程建成使用。成功承办了中央电视台“芜湖月?中华情”中秋晚会、第十五届全国青少年车辆模型教育总决赛、首届全国大学生空手道锦标赛。我市运动健儿再创佳绩，在广州亚运会上获得2块金牌1块铜牌，在省第十二届运动会上夺得总分第二名。计生工作取得新进展，在全省率先开展免费孕前优生健康检查，人口出生率8‰。第六次全国人口普查进展顺利。适时出台了稳定粮油、蔬菜副食品价格的政策措施，完善市级商品应急储备体系，新建、改建市区菜市场12个，对困难群众发放物价补贴1438万元。完成了“五五”普法工作，扎实推进依法治市，深入开展“双百日维稳攻坚行动”，完善信访工作制度，排查化解社会矛盾。公开挂牌整改安全生产重大隐患25个，安全生产形势总体稳定。加强社会治安综合治理，保持严打高压态势，群众安全感进一步提高。认真做好双拥优抚安置工作，国防后备力量建设进一步加强，我市被评为全国人民防空先进城市。民族、宗教、气象、档案、地方志、老龄、残疾人等事业取得新进步。福利彩票慈善助学项目荣获中华慈善奖。镜湖区被评为全国养老社会化服务示范区、全国社区服务先进单位。繁昌县、镜湖区被评为全国残疾人社区康复示范县区。

（七）加强政府自身建设，提高服务能力和水平。扎实开展“创先争优”活动，大力加强干部队伍学习培训，大力倡导勤勉务实的优良作风，大力推行“一线工作法”，努力增强推动科学发展的本领。全面推进依法行政，自觉接受人大监督和政协民主监督，积极听取各民主党派、工商联、无党派人士的意见和建议，主动加强与工会、共青团、妇联等人民团体的密切联系。全年办理人大议案2件、建议105件和政协提案361件，办复率100%；回复和办理人民网、中安在线网和市民心声网民留言、咨询7280多条，回复率100%。实现审批项目在线申报、“一窗式”综合受理，建立项目审批“快捷通道”机制，全面推广应用电子政务统一办公平台，努力提高办事效率。深入开展“万人千企评议政风行风”活动，强化服务对象监督，不断改进工作作风。坚持廉洁从政，加强制度建设，严格政府性资金审计，强化财政资金监管，厉行勤俭节约，推行“阳光村务”，开展“小金库”、工程建设领域突出问题等专项治理，廉政建设取得积极成效。

2010年是实施“十一五”规划的最后一年。回首极不平凡的“十一五”，我们有效应对了百年一遇的国际金融危机的严重冲击，成功战胜了多重自然灾害的重大挑战，谋划实施了一批多年想办而没有条件办的大事要事，全市经济社会发展取得了巨大成就。

这五年，是综合实力大幅提升的五年。主要经济指标总量跃居全省第二位。地区生产总值跨过6个百亿元台阶，年均增长16.1%，为改革开放以来增长最快的时期之一。累计固定资产投资3430亿元，是“九五”、“十五”十年的3.4倍。规模工业企业由552家增加到1864家，4家企业产值超百亿元。财政收入跨过2个百亿元台阶，年均增长24.3%。

这五年，是结构调整成效显著的五年。工业化率由46.5%提升到58.9%，三个支柱产业对工业增长的贡献率达到69.8%。高新技术产业增加值占地区生产总值的比重由14.1%提升到24.9%。粮食产量稳定在84万吨左右，比“十五”末增产7万吨。非公有制经济比重由56.9%提升到62%。

这五年，是城乡面貌日新月异的五年。城市基础设施建设投入480亿元，是“十五”时期的9.6倍。市区建成区面积由95平方公里增加到135平方公里、人口由73万增加到112万。城镇化率由54.3%上升到68%。城镇道路由756公里增加到1790公里。统调机组发电装机容量由50万千瓦增加到198万千瓦。完成了万亩圩口堤防达标工程。新增污水处理能力43万吨。新建滨江公园，扩建赭山公园，完成保兴垾整治一期工程，新增绿地1150万平方米。高速铁路、火车站改扩建、芜申运河、青弋江分洪道、响水涧抽水蓄能电站等一大批打基础、管长远的重大项目获批建设。

这五年，是改革开放成果丰硕的五年。农村综合改革取得成效，国企改革进一步深化，金融改革和创新不断加快。事业单位改革在全省率先实施，医药卫生体制改革取得阶段性成果，文化体制改革进入全国先进行列。完成了公务员津补贴和部分事业单位绩效工资改革。创新行政和财政管理体制机制，简政放权，激发活力。对外开放成效显著，五年累计利用外资27.7亿美元、利用内资2689亿元，分别是“十五”期间的3.3倍、6.3倍。获得进出口经营权的企业达1140家，净增867家，进出口总额年均递增31.5%。我市被列为成功开拓中国特色发展之路的全国18个典型地区之一、皖江城市带承接产业转移示范区的核心城市、合芜蚌自主创新综合试验区的中心城市，城市影响力、竞争力明显提升。

这五年，是人民生活明显改善的五年。实施了53项民生工程，累计投入102亿元。新增就业27.3万人。城市居民人均可支配收入、农民人均纯收入年均分别增长14.1%和13.2%，比全省平均水平分别高3100元、2400元左右。市科技馆、图书馆、文化馆建成投入使用。医疗、卫生、教育等社会事业全面进步。社会保障体系基本建立，保障水平明显提升，企业退休人员养老金月人均增加726元。农村五保户集中供养率由20%提升到51%。城市人均住房建筑面积由25平方米增加到30平方米，农村人均生活用房面积由31.9平方米增加到37.5平方米，机动车保有量由7.3万辆增加到24.5万辆，居民人均储蓄存款由0.9万元增加到2.06万元。

“十一五”发展取得的成绩来之不易，积累的经验更是弥足珍贵，今后我们必须倍加珍视并要长期坚持。一要始终坚持党的领导，把中央和省委、省政府决策部署与我市实际紧密结合起来，抢抓机遇，创造性地开展工作，积极应对各种挑战，不断开拓具有芜湖特色的科学发展之路。二要始终坚持推进自主创新，不断加强以企业为主体的创新体系建设，培育一批在全国“叫得响”的创新典型。三要始终坚持弘扬创新文化，崇尚创新、宽容失败、支持冒险、鼓励冒尖，营造尊重、爱护、宽容、保护企业家的良好氛围，让一切创新创业的源泉充分涌流、竞相迸发。四要始终坚持与时俱进、解放思想，不断深化重点领域改革，坚决破除影响科学发展的体制机制障碍，优化发展环境，增强发展动力和活力。五要始终坚持对外开放，把招商引资作为经济工作的重中之重，把推进项目建设作为经济工作的生命线，不断提高对外开放合作层次和水平。六要始终坚持统筹兼顾，注重生态环保、招商引资并举，注重“好”字当头、又好又快；注重以城带乡，加快城乡一体化发展。七要始终坚持以人为本，强化群众观念，把更多的时间精力用在帮助基层解决实际问题上，把更多的财力物力用在经济社会发展和人民生活水平提高上，确保社会和谐稳定。八要始终坚持依法行政，强化社会管理和公共服务，着力转变政府职能，提高政府效能，建设法治政府。

各位代表！

“十一五”的发展，为全面建设小康社会奠定了坚实基础，在芜湖发展史上谱写了辉煌篇章。这是中共芜湖市委统揽全局、坚强领导的结果，是市人大代表充分行使职权、市政协委员积极参政议政的结果，是全市人民团结奋进、社会各界合力共建的结果。在这里，我谨代表市人民政府，向全体人大代表、政协委员，向全市广大工人、农民、知识分子、干部、驻芜解放军指战员、武警官兵和政法干警，向各民主党派、工商联、各人民团体、无党派人士、离退休老同志和社会各界人士，向在我市创业的投资者、建设者，向为芜湖发展作出积极贡献的中央、省直各部门和海内外友好人士，表示衷心的感谢和崇高的敬意！

各位代表！

回首极不平凡的“十一五”，我们也清醒地看到，前进道路上还存在不少困难和问题。主要是：经济规模不够大、产业结构不够优、竞争力不够强的市情没有根本改变。战略性新兴产业规模较小，企业自主创新能力还不够强。第三产业发展相对不足，农业现代化水平亟待提高。土地、资源、环境制约日益增强，节能减排任务依然艰巨。统筹城乡发展步伐尚需加快，城市规划建设管理水平有待提升。以改善民生为重点的社会建设仍需进一步加强，行政效能和服务水平还需进一步提高。少数干部群众观念淡薄，存在形式主义、官僚主义、享乐主义，腐败现象时有发生。对此，我们一定高度重视，采取更加有力措施，认真加以解决，绝不辜负全市人民的期望和重托！

二、科学发展、率先崛起的锦绣前程

“十二五”时期，是我市全面建设小康社会的关键期，也是加快转变经济发展方式，加速推进工业化、城市化、农业现代化的战略机遇期，是率先崛起的黄金发展期。未来五年加快发展的机遇前所未有，是大有作为、大有可为的五年。作为皖江城市带承接产业转移示范区核心城市和合芜蚌自主创新综合试验区中心城市，政策叠加效应日益显现；改革开放不断深化，发展活力和动力日益强劲；经济实力不断提升，发展后劲日益增强；交通和产业基础不断完善，区域经济中心地位日益提高。但同时我们必须清醒地看到，未来五年的挑战也是前所未有，世界经济复杂多变，区域竞争日趋激烈，资源环境约束加强，转型发展任重道远。我们必须进一步增强机遇意识、忧患意识和责任意识，把握发展规律，利用各种有利条件和积极因素，主动应对挑战，聚精会神抓建设，一心一意谋崛起，努力实现经济社会更好更快发展。

“十二五”经济社会发展的指导思想是：以邓小平理论和“三个代表”重要思想为指导，以科学发展为主题，以全面转型、率先崛起、富民强市为主线，全面实施“创新驱动、产业强市、统筹城乡、开放合作、和谐发展”五大发展战略，着力推进皖江城市带承接产业转移示范区、合芜蚌自主创新综合试验区建设，促进产业高端化、城市现代化、环境生态化，建设创新芜湖、优美芜湖、和谐芜湖、幸福芜湖，立足全省次中心城市定位，把芜湖建设成为全国重要的先进制造业基地、综合交通枢纽、现代物流中心、创新型城市和长江流域具有重要影响的现代化大城市。

综合考虑未来发展趋势和条件，努力实现“12861”发展目标。“十二五”期间，累计完成全社会固定资产投资1万亿元；到2015年，地区生产总值达2800亿元，工业销售收入达6000亿元，人均地区生产总值达10万元，确保城乡居民收入比2010年翻一番。

突出把握好以下五个方面：

第一，实施创新驱动战略，增强核心竞争优势。推进自主创新综合试验区建设，全面提升科技创新能力。完善财政投入稳定增长机制，落实鼓励研发的财税政策，健全以企业为主体、以市场为导向、产学研相结合的技术创新体系。建立部门联动机制，实施一批重大科技项目。树立人才资源是第一资源的理念，加强创新创业人才队伍建设。增强企业自主创新能力，实施“小巨人”企业培育计划，加大企业上市力度，引导企业加大研发投入。放大产学研合作效应，推动科技成果产业化。支持优势企业加强技术标准、体系标准的研究和制定。加快创新平台建设，推进国家汽车零部件产品质量监督检验中心等公共服务平台、技术平台建设，为企业创新提供技术和服务支撑。实施知识产权战略，加强商标品牌建设。力争到2015年，高新技术产业增加值占地区生产总值的比重达到33%。

第二，实施产业强市战略，推动经济结构转型升级。坚持走新型工业化道路，集中力量发展壮大汽车及零部件、材料、家用电器三个支柱产业，把芜湖建设成为全国重要的汽车、新型材料、家电生产基地；加快发展节能环保、装备制造、电子信息、生物医药四个战略性新兴产业，实施一批重大项目，形成经济发展的先导产业和支柱产业；改造提升能源、化工、船舶制造、纺织服装、烟草食品等传统产业。到2015年，努力打造汽车、材料、光电、家电4个销售收入超千亿元产业。把推动服务业大发展作为产业结构优化升级的战略重点，优先发展金融、现代物流、文化创意、服务外包和旅游等现代服务业，大力发展中介服务业、商贸业、会展业、养老和家庭服务业，积极培育总部经济，加快建设一批特色鲜明的专业化大市场和服务业集聚区，不断推进第三产业规模扩张、结构优化和竞争力提升。到2015年，力争第三产业增加值占地区生产总值的比重达到35%。坚持用工业化理念发展都市型现代农业，改善农业生产条件，优化特色农产品区域布局，促进农业生产经营专业化、标准化、规模化、集约化。大力发展循环经济，加快低碳技术研发和应用，推行清洁生产，倡导绿色消费。

第三，实施统筹城乡战略，建设现代化大城市。按照规划统领、市域一体、组团发展、彰显特色的发展思路，提升城市综合承载力、集聚力、辐射力和带动力，力争到2015年城镇化率达到75%。构建组团式城市发展格局，推进中心城区“东扩、南进、中提升”，发展湾沚、繁阳、籍山三个城市副中心，建设特色中心镇。构建现代交通网络，建成宁安城际铁路、京福客运专线、商杭客运专线、皖赣新建双线、火车站改扩建、芜雁高速公路、长江公路二桥、芜申运河等重大工程。加快城乡供水、供电、供气、污水垃圾处理、水利等基础设施建设，完成青弋江分洪道和县城防洪工程。加大环卫、市政、园林等行业改革力度，实现数字化城市管理全覆盖。坚持公共交通优先发展，优化线网结构，完善场站建设，提升服务质量。促进房地产业平稳健康发展，逐步形成保障性住房体系和商品房体系。加快“智慧城市”建设，推进国民经济和社会信息化。在确保农民利益有保障和耕地数量不减少、质量不下降的前提下，以土地整治整村推进为突破口，统筹城乡协调发展。逐步实施一元化户籍制度，把更多符合落户条件的农业人口逐步转为城镇居民。推进主体功能区建设，逐步形成人口、经济、资源环境相协调的国土空间开发格局。加强生态建设和环境保护，节约集约利用能源资源，加强水、大气、农村面源的污染防治，加强矿山地质环境治理恢复，开展植树造林和园林绿化，保护森林公园、湿地、文化遗产等自然和人文景观，力争市区绿化覆盖率达41%，全市森林覆盖率达23%，创建国家园林城市。

第四，实施开放合作战略，大力发展外向型经济。充分利用国际国内两个市场、两种资源，实施“引进来”与“走出去”战略，构建全方位、宽领域、多层次的对外开放合作新格局。推进皖江城市带承接产业转移示范区建设，按照“三年见成效，五年大发展”的要求，利用先行先试权，突破体制机制障碍，营造良好投资环境。加大招商引资力度，抓好战略性新兴产业、现代服务业招商，新上一批重大项目，在引进世界500强企业、行业龙头企业和上市公司上取得更大成效。深化区域合作，推进芜马同城化、跨江联动发展，全面参与泛长三角发展分工合作。积极加强友城交流，深化与亳州等市的结对合作。

第五，实施和谐发展战略，提升人民幸福指数。健全覆盖城乡的基本公共服务体系，推进基本公共服务均等化。大力实施民生工程，着力解决群众切身利益问题。千方百计扩大就业，扎实推进城乡统筹就业全国试点、国家级创业型城市试点工作。大力发展民营经济，鼓励全民创业。健全社会保障体系，提高城乡居民养老、医疗和最低生活保障水平。加快基本养老服务体系建设，完善社会救助体系，大力发展福利慈善事业。加快建立现代教育体系，大力发展学前教育，率先普及15年基础教育，打造长三角地区具有重要影响的高等教育和职业教育强市。完善公共卫生和基本医疗服务体系，深化医药卫生体制改革，推进基层医疗卫生服务机构标准化建设，完善药品供应保障体系，加强血吸虫病等重大传染病防治，做强三大医疗集团，鼓励社会资本创办特色、高端医疗技术服务机构，支持中医药事业发展。推动文化大繁荣大发展，坚持社会主义先进文化前进方向，深化文化体制改革，繁荣文艺事业，完善文化、广播电视等基础设施，构建覆盖城乡的公共文化服务体系。加强精神文明建设，争创全国文明城市。加强体育基础设施建设，深入开展全民健身运动，提高全民身体素质。坚持计划生育基本国策，全面做好人口工作。完善对妇女、儿童、老年人、残疾人等群体的社会服务，做好民族、宗教工作。全面加强社会管理，健全党委领导、政府负责、社会协同、公众参与的社会管理格局。开展“六五”法制宣传教育，坚持依法治市，完善基层管理和服务体系，加强社会治安综合治理，维护社会和谐稳定。

三、全面转型、富民强市的崭新征程

2011年是实施“十二五”规划的开局之年，做好今年的各项工作，意义重大。综合考虑，今年经济社会发展的主要预期目标是：地区生产总值增长15%以上，财政收入同步增长，全社会固定资产投资增长25%以上，全部工业销售收入增长25%，社会消费品零售总额增长18%，进出口总额增长15%，城镇居民人均可支配收入、农民人均纯收入分别增长13.5%，城镇化率提高2个百分点，高新技术产业增加值占地区生产总值比重提高2个百分点，现代服务业增加值占第三产业比重达36%，新兴产业实现增加值增长25%，城镇新增就业6万人，城镇登记失业率控制在4.5%以内，人口出生率控制在8‰以内，节能减排完成省政府下达目标。

为实现上述目标，我们将着力抓好以下八个方面的工作：

（一）全力保持经济持续快速增长

力争更好更快的发展，是今年经济工作的首要任务。坚持市领导分工联系重大项目制度，加强项目跟踪服务，加快项目建设。围绕壮大三大支柱产业，加快推进奇瑞轿车五厂和新能源汽车、集瑞联合重工和玉柴联合动力二期、格力电器、新兴铸管三山基地、芜湖海螺水泥三期、美的出口空调二期等重点项目建设。围绕发展四大战略性新兴产业，加快推进三安光电和德豪润达光电二期、信义超薄玻璃等重点项目早竣工、早投产。改造提升传统产业，推进服装品牌创建，发展船舶核心装备和关键零部件产业，推进恒安纸业、都宝卷烟、双汇食品等项目建设。鼓励企业实施技改，提升工艺技术、装备水平和产品档次。积极引进域外金融机构，扶持地方法人金融机构，鼓励小额贷款、担保、典当、风险投资机构发展，引导设立金融租赁公司、财务公司、信托公司、消费金融公司，创新金融服务，扩大信贷规模。支持企业直接融资，加强企业上市工作，力争申报3家以上。完善港航基础设施，加快建设南翔万商、裕溪口煤炭储配中心、方村物流基地等重点物流项目。积极发展文化创意产业，加快华强文化科技产业园二期、鸠江文化创意产业园等项目建设。以方特两个主题公园为龙头，加快芜湖古城等项目和星级宾馆建设，整合旅游资源，推动旅游产业升级。加快推进弋江区服务外包产业园二期建设，大力发展总部经济，加快区域性大市场建设，积极培育会展经济。推进南陵现代农业示范区和大浦农业科技园区建设，推广超级稻丰产工程，实施优质油菜生产基地项目，加强“菜篮子”工程建设，发展无公害农产品、绿色食品和有机农产品，培育农业标准园、休闲生态观光园。成立农业担保公司，重点支持龙头企业发展和农民创业，培育一批农民专业示范合作社，壮大一批农业产业化龙头企业。大力拓展消费需求，落实扩大内需政策，培育旅游、文化、体育等消费热点。统筹调度市级资金，支持县区经济发展。

（二）进一步加快城市现代化建设步伐

把加快城市现代化作为全面转型的战略重点，摆在更加突出的位置。一是坚持规划引领。完成城市总体规划（2010－2030年）修编，编制完善给排水、污水处理、公交、燃气、环卫、绿地系统、水环境保护、旅游、商业网点、城市轨道交通等专业专项规划，开展市域综合交通等专题研究。二是推进城市大建设。加快铁路、芜申运河、芜雁高速公路工程建设。力争商杭客运专线、芜湖长江公路二桥项目获批建设。加快芜屯路清水河公路桥建设，开工建设三荻路漳河大桥和马园河桥。加快县乡道路升级改造、危桥改造、渡改桥和农村公路安保工程建设。实施城市畅通工程，新建中江大道南延段等14条道路，续建长江北路延伸段等一批改造工程，建设人行天桥、地下通道和停车场，新增和更新公交车378辆。开工建设利民路水厂二期、三山自来水厂。新建和改造一批加气站。深入实施“大绿化”工程，加快建设和改造龙窝湖公园、凤鸣湖公园、芦花塘公园、大阳垾湿地公园、汀棠公园、神山森林公园等一批市、区级公园，新增绿地面积450万平方米，力争建成国家园林城市。开工建设青弋江分洪道工程，实施农村安全饮水、水库除险加固、重点泵站建设、河流湖泊综合治理等工程，加强农田水利基本建设，增强防汛抗旱能力。推进农村信息化，为广大农民提供更多更有效的信息服务。三是强化城市管理。开展市容整治提升年活动，健全工作机制，实施美化亮化工程，推进老旧小区整治、沿街立面改造，提高公共设施建设和管理水平。严控违法建设，强化渣土管理，整顿交通秩序。扩大市区数字化城管覆盖面，推进三县数字化城管建设，提高管理效率。

（三）大力推进创新型城市建设

深入推进国家技术创新工程试点和合芜蚌自主创新综合试验区建设。做好新一轮全国科技进步先进市考核工作，争取列入国家科技金融创新试点城市。力争新增高新技术企业50家、高新技术产品100个，高新技术产业总产值增长30%。完善科技创新平台，加快创业中心、汽车电子及关键零部件企业孵化器、高新区大学科技园建设，力争申报省级以上研发机构20家，新增科技企业孵化器5万平方米。健全“科技路路通”芜湖分中心服务功能，新建服务站10个。力争专利申请量5700件、授权量2700件。争取建立国家专利技术（芜湖）展示交易中心，促进专利技术转化。深化产学研合作，引进高校、科研院所来芜设立研发和技术转移机构。支持留学人员芜湖创业园升级为省部共建园。把人才资源开发利用放在优先位置，加大招才引智和本地创新型人才培育力度，营造尊重人才的社会环境，促进优秀人才脱颖而出。

（四）突出抓好重点领域和关键环节改革

面对新形势新任务，我们必须以更大的决心和勇气，进一步推进改革。一是深化医药卫生体制改革。深化公立医院改革，建立公立医院与城乡基层医疗卫生机构的分工协作机制，改革医院收费结算方式，加快医院信息化建设和体制机制改革。二是深化事业单位人事分配制度改革。转换用人机制，健全全员聘用和岗位管理制度，稳步推进事业单位绩效工资改革。三是深化国有企业改革。建立国有企业绩效考核办法，完善法人治理结构。创新国有资产运营管理体制，提高运营能力。四是深化财政改革。调整城镇土地使用税征管和奖励机制，促进土地集约节约利用。深化国库集中支付改革，调整市直机关财务核算体制。扩大预算支出绩效考评范围。科学编制预算，整合财政资金，集中财力办大事。五是深化社会保障制度改革。完善各项社会保险制度，积极探索城镇职工养老保险、新农保、城乡居民养老保险衔接办法，畅通养老保险异地转移通道。六是深化统计制度和方法改革，建立科学的统计审核体系。七是大力推进创业富民。市、县区设立创业富民专项扶持资金，以贴息、补贴、奖励、风险补偿等方式，大力支持民营经济发展，积极引导扶持全民创业。

（五）全面推动皖江城市带承接产业转移示范区建设

始终牢记发展是第一要务、招商是重中之重，持之以恒地保持扩大对外开放、承接产业转移的高涨热情和强劲势头。完善功能配套，高起点建设专业园区，强力推进经济技术开发区“二次创业”。加快长江大桥开发区循环经济园区基础设施建设，加强综合保税区报批工作，尽快形成承接产业转移的新优势。以高新技术产业开发区升格国家级为契机，调整优化产业结构，壮大高新技术产业规模。大力提升省级开发区承接产业转移的层次和水平，着力在引进大项目和大企业上取得更大突破。加快建设一批标准厂房、蓝领公寓，提高开发区土地利用率，优化开发区生活配套环境。创新招商方式，发挥国内外知名商会、协会作用，提高招商小分队专业化水平，强化招商项目全程跟踪服务，增强招商工作的针对性和有效性。更加重视引进技术、人才和研发中心，加强与中央企业、全国知名民营企业、世界500强外资企业的对接联系，力求取得更大进展。

（六）努力建设资源节约型、环境友好型社会

着力缓解日趋强化的资源环境约束，增强可持续发展能力，将是我们长期的重大任务。一是要更加突出土地集约节约利用。扎实推进农村土地整村整治工作，加强耕地保护，拓展发展空间。对土地投资强度、单位产出率、建筑容积率、土地供应率等指标实行最严格的考核，提高土地利用效率。对省级开发区以外的工业项目投资，实行市级审批，从严控制。继续清理土地出让合同，对承诺事项进行对照检查，纠正违约行为，防止占地不用。二是要更加重视节能减排。层层落实节能减排工作责任制，强化目标考核。加快节能新技术、新工艺、新产品的推广应用，健全重点耗能企业监控机制，推进建筑业、交通运输业、商业、公共机构等领域的节能减排。大力发展循环经济，认真谋划资源节约和综合利用项目，做好奇瑞、海螺、新兴铸管等循环经济示范工作。加快滨江、城东、芜湖县、繁昌县污水处理项目建设，完善配套管网，提高污水处理率，扎实推进主要污染物减排。从今年开始，3年内基本完成市区所有老旧居民小区雨污分流改造。三是要更加注重生态环境建设。强化环境监管，实施严格的饮用水源和地表水域保护制度。整治环境污染突出问题，巩固保兴垾整治、小炉窑烟尘整治以及“三湖”（银湖、凤鸣湖、汀塘）整治成果，启动板城垾、保兴垾上游及支流整治工程。开展农村主要沟渠湖塘的清淤疏浚，打造一批“河畅、水清、岸绿、景美”的水环境典型。大力开展爱国卫生运动，加强农村生活污水、垃圾处理，完善城乡垃圾收集处置一体化网络。严控山体开挖、矿山开采，并形成严格的报批制度。深入开展绿色系列创建活动，建设沿江、沿湖抑螺防病林，推进社会化办林业，积极争创国家级和省级生态县。

（七）高度重视解决民生问题

把保障和改善民生，始终作为政府工作的出发点和落脚点。一是大力实施民生工程。以完善基本公共服务、创新社会管理为重点，解决群众关切的重大问题，研究改善民生的长效机制，不断增进群众福祉。在实施省民生工程基础上，新增城市公共交通建设、学前教育发展2项，并扩展增加血吸虫病防治、“菜篮子”工程等内容，共计划实施39项民生工程、安排资金38.3亿元。二是更加重视促进城乡居民收入提高，大力实施城乡居民收入“五年倍增计划”。进一步加强城乡公共就业服务，完善农民工就业培训机制，对困难群体实施就业援助，大力扶持城乡居民就业、创业，推行工资集体协商制度，增加居民的工资性、经营性和财产性收入。进一步完善社会保障体系，加强困难群体救助，逐步提高城乡低保标准和被征地农民养老保障标准，建立物价上涨与财政补贴联动机制，保障低收入家庭基本生活，增加居民转移性收入。三是保持房地产市场健康发展。加大保障性安居工程建设力度，建设拆迁安置房420万平方米，新开工建设保障性住房110万平方米，改造棚户区1.2万户。出台支持房地产开发政策，增强投资者、消费者信心，增加市场供应，加大对外营销力度。扩大住房公积金制度覆盖面，支持职工改善住房条件。四是做好教育、卫生、文化等工作。制定学前教育三年行动计划，实行政府主导、社会参与、公办民办并举，切实解决“入园难”问题。大力发展职业教育，加快职教园区建设。积极支持在芜高校建设，鼓励高校融入地方经济社会发展。扩大基本公共卫生服务覆盖面，促进公共卫生服务均等化。开展专项整治，保障食品药品安全。加强出生缺陷干预，提高计划生育综合服务水平。加快市规划馆和博物馆、县区图书馆和文化馆等建设步伐，丰富群众文化生活。开工建设奥林匹克公园二期工程，推进公共机构体育设施社会化服务，大力发展体育事业和体育产业。编制和实施2011－2020年妇女和儿童发展规划。重视民族、宗教工作，关爱留守流动儿童，发展老龄、残疾人事业。

（八）着力维护社会和谐稳定

深入开展群众性文明创建活动，提升全民素质，积极争创全国文明城市。启动“六五”法制宣传教育工作，加强法律援助、律师、公证和人民调解工作，提高全社会法制化管理水平。开展第八届村委会换届选举，加强城乡和谐社区建设，推广社区信息化试点，改进社区服务。高度关注和解决征地拆迁的遗留问题，严格执行相关政策，切实维护和保障人民群众的合法权益。创新信访机制，拓宽沟通渠道，引导群众依法表达利益诉求，排查化解社会矛盾纠纷。落实安全生产责任，加强交通、建筑、消防等重点领域安全专项整治。加强应急救援队伍建设，加大装备投入，提高保障公共安全和处理突发事件的能力。支持部队建设，重视国防教育、国防动员、民兵预备役和人民防空工作，争创新一轮全国双拥模范城。加强社会治安防控体系建设，严厉打击违法犯罪活动，注重从源头上预防和减少社会矛盾，维护社会稳定。

各位代表！

全面完成今年和“十二五”时期各项任务，对政府自身建设提出了更高要求。我们将进一步深入开展“创先争优”、建设学习型机关活动，不断提高谋划、推动科学发展的能力，力戒骄傲自满，力求各项工作争上游、争一流。进一步做好新形势下的群众工作，牢固树立群众观点，始终站稳群众立场，创新群众工作方式，健全群众工作制度，维护群众切身利益。进一步提高机关效能，整合行政资源，理顺部门职能关系，提高办事效率，改进服务态度，健全科学的考核评价体系，坚决治懒治庸。进一步推进依法行政，坚持科学民主决策，自觉接受人大监督和政协民主监督，认真办理人大议案、建议和政协建议案、提案，充分听取各民主党派、工商联、无党派人士及各人民团体的意见。进一步加强政务公开和网络问政，继续开展“万人千企评议政风行风”活动，充分发挥服务对象、社会和媒体的监督作用，加强行政监察和审计监督，加大问责问效力度。进一步优化政治生态，精诚团结，同心协力，维护大局，始终保持昂扬向上的精神状态，始终保持用心用力的工作激情，始终保持勤勉尽责的务实作风，坚持推进科学发展不折腾、不懈怠、不动摇。进一步加强廉政建设，落实廉政责任制，加强廉政教育，强化制度建设，健全惩治和预防腐败体系，坚持勤俭节约，力戒奢侈浪费，着力打造廉洁政府。

各位代表！

回顾成就辉煌的奋进历程，我们无比自豪；展望无限美好的锦绣前程，我们心潮澎湃；踏上跃马扬鞭的崭新征程，我们豪情满怀。让我们在中共芜湖市委的坚强领导下，紧紧依靠和团结全市人民，励精图治，锐意进取，倍道兼程，奋发有为，努力开创科学发展新局面，以优异成绩向中国共产党成立90周年献礼！
